# A review of targeted drug delivery with antibody-drug complexes

**DOI:** 10.1016/j.jpet.2025.103732

**Published:** 2025-12-30

**Authors:** Dhruv Sanjanwala, Ying Meng, Zhiling Guo, Brandon Bordeau

**Affiliations:** Department of Pharmaceutical Sciences, College of Pharmacy, University of Michigan, Ann Arbor, Michigan, USA

**Keywords:** Antibody-drug conjugate, Antibody-drug complex, Targeted drug delivery, Antibody engineering, Bispecific antibody, Degrader-antibody conjugate

## Abstract

Monoclonal antibodies are a versatile platform for targeted drug delivery. Their high specificity and favorable pharmacokinetics allow for selective drug delivery to targeted cells. A primary drug delivery application is antibody-drug conjugates (ADCs), which combine monoclonal antibodies with cytotoxic payloads via covalent linkers. While ADCs have shown remarkable clinical success, several limitations remain, including complex conjugation chemistries, heterogeneity in drug-to-antibody ratios, exposed hydrophobic patches, and off-target payload release, which can result in systemic toxicity. To complement existing ADC platforms and mitigate some of these issues, alternative formats referred to as antibody-drug complexes (ADCx) have been developed. ADCx are generated by forming reversible, high-affinity complexes between antibodies and drugs or preformed drug conjugates. This review discusses the ADCx formats reported to date, focusing on the unique advantages and potential limitations of each format.

**Significance Statement:**

Antibody-drug complexes offer a modular, noncovalent alternative to traditional antibody-drug conjugates. This review comprehensively evaluates antibody-drug complex formats, highlighting their potential to expand the utility of antibody-based drug delivery for next-generation therapeutics.

## 1 Introduction

Monoclonal antibodies have long circulation half-lives and can bind antigens with high affinity and high selectivity. By engaging with specific cell surface antigens, antibodies can facilitate receptor-mediated internalization and lysosomal trafficking, thereby enabling intracellular delivery of therapeutic cargo.^1^ These attributes have led to the use of monoclonal antibodies as a delivery vehicle for cytotoxic drugs,^2^ radionuclides,^3^ cytokines,^4^ nucleotide therapies,^5^ protein toxins,^6^ and imaging probes.^7^

To date, significant clinical success in antibody-mediated drug delivery has been achieved with antibody-drug conjugates (ADCs). ADCs consist of a monoclonal antibody bridged to a cytotoxic drug via a chemical linker. Currently, 14 ADCs are approved by the Food and Drug Administration (FDA), and an additional 198 are in clinical trials.^8^, ^9^, ^10^ Recently, ADC use has expanded to earlier stages and a wider range of tumor types.^11^, ^12^, ^13^ For example, trastuzumab deruxtecan (Enhertu), an ADC targeting the human epidermal growth factor receptor (HER)2 receptor, was first approved by the FDA in 2019 for the treatment of unresectable or metastatic HER2^+^ breast cancer (after patients have received 2 or more anti-HER2 therapies).^14^ Between 2022 and 2025, its indications were expanded by the FDA to include recurrent HER2^+^ metastatic breast cancer, HER2-low or HER2-ultralow metastatic breast cancer, and any advanced HER2^+^ solid tumors (including lung, colorectal, and gastric cancers).^12^^,^^15^, ^16^, ^17^, ^18^, ^19^, ^20^, ^21^, ^22^ Ten of the 14 ADCs approved to date received regulatory approval since 2019, marking a golden era in ADC development. Further, ADCs are emerging options for noncancer indications with ADCs entering clinical trials for the treatment of infectious diseases and autoimmune disorders.^23^^,^^24^ Despite the clinical success of ADCs, the need for a covalent linker has well-appreciated limitations.^25^, ^26^, ^27^, ^28^, ^29^, ^30^ These limitations include complex conjugation chemistries,^26^^,^^31^ drug-to-antibody ratio (DAR) heterogeneity,^26^^,^^32^ increased aggregation due to exposed hydrophobic payloads,^33^ and premature linker catabolism,^34^, ^35^, ^36^, ^37^ which can lead to systemic toxicities.^30^

As an alternative to traditional ADCs, several groups have developed noncovalent antibody-drug complexes (ADCx). A defining feature of ADCx is the absence of a covalent linker between the antibody and the drug. Instead, the drug may bind directly to the antibody or indirectly via a secondary binding partner. Depending on the format, ADCx offers several potential advantages. First, direct antibody-drug binding eliminates the need for chemical modification, allowing for a simple, 1-step complex formation that minimizes the risk of impairing either the antibody or the payload’s function. Second, ADCx enables precise control over the DAR, improving product uniformity. Additionally, specific ADCx formats eliminate the risk of off-target linker cleavage, a standard limitation of traditional ADCs that can lead to premature payload release and systemic toxicity.^30^^,^^34^, ^35^, ^36^, ^37^ In specific ADCx designs, the payload is sequestered within the antibody’s binding site, potentially mitigating the increased clearance that is observed for ADCs with exposed hydrophobic payloads. Currently, there are no FDA-approved ADCx therapies, and research on ADCx formats has been limited relative to ADCs. However, given the unique characteristics of ADCx, there are emerging use cases that are likely to increase interest in noncovalent antibody-based drug delivery. Like ADCs, ADCx can be used to deliver many payloads for the treatment of a variety of diseases.

**Fig. 1:**
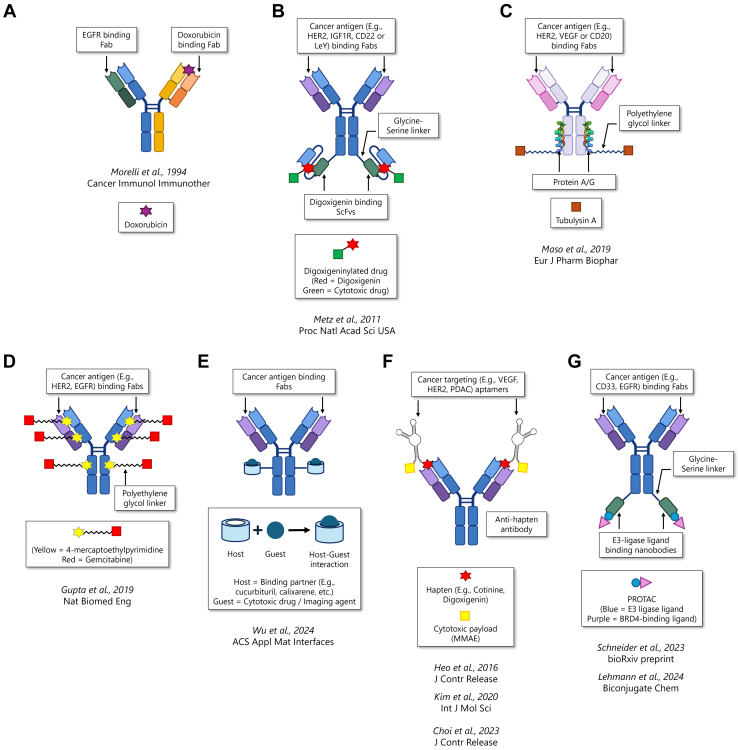
Representative strategies for generating noncovalent ADCx: (A) Bispecific antibodies engineered to simultaneously recognize a tumor-associated antigen (eg, EGFR) and a chemotherapeutic payload (eg, doxorubicin); (B) Bispecific antibodies comprising tumor antigen-binding Fab regions and digoxigenin (Dig)-binding scFvs appended to their heavy chain C-termini. Cytotoxic payloads digoxigeninylated using NHS ester chemistry noncovalently bind to the scFvs, forming an ADCx. (C) Protein A/G conjugated to a cytotoxic drug (eg, tubulysin A) via a PEG linker noncovalently binds the Fc region of tumor-targeting IgGs. (D) MEP-conjugated cytotoxics (eg, gemcitabine) form noncovalent complexes with conserved pockets on both Fab and Fc regions of IgG, generating MAGNET ADCs. (E) Bispecific antibodies engineered by fusing nanobodies that recognize E3-ligase ligands (eg, VH032) to the C-terminus of a targeting antibody. These shuttle PROTAC molecules to the tumor. (F) Oligobody/DOligobody strategy: aptamers specific to tumor antigens (eg, HER2 and PDAC) are conjugated to a cytotoxic drug and a hapten (eg, digoxigenin or cotinine). This aptamer-drug-hapten construct forms a noncovalent complex with a universal antihapten antibody for targeted delivery. (G) Supramolecular ADCs involving host-guest interactions, where the antibody is conjugated to a macrocyclic host (eg, cucurbituril and calixarene), which binds various cytotoxic or imaging guest molecules to form a stable yet modular delivery complex (Made using BioRender.com) Fab, antigen-binding fragment; VEGF, vascular endothelial growth factor.

In this review, we provide a comprehensive and critical overview of the current strategies used to create ADCx. We evaluate the available data for each approach, highlighting the opportunities, challenges, and future directions for noncovalent antibody-based drug delivery systems. Each format is schematically illustrated in Fig. 1 and summarized in Table 1.^38^, ^39^, ^40^, ^41^, ^42^, ^43^, ^44^, ^45^, ^46^

**Table 1:** The approaches used to develop antibody-drug complexes, along with the binding affinities, IC_50_ values, DARs, and pharmacokinetics achieved using each modality

Approach	Figure	Description	Affinity and DAR	Results
Bispecific antidrug anticancer ADCx^38^ (Patent: EP0439048A2)	Figure 1A	Bivalent bispecific IgG antibody targeting doxorubicin and EGFR. Antibody was used as a pretreatment for modifying doxorubicin biodistribution.	K_D_ (for doxorubicin): 17 nM DAR: 1	In vitro: IC_50_ in EGFR^+^ cells was ∼8× lower than that in EGFR^−^ cells In vivo biodistribution/PK: Doxorubicin accumulation was ∼1.2× higher in the tumor and ∼1.5× lower in the intestine, in mice pre-treated with the bispecific antibody. In vivo toxicity: In mice, pretreatment with the bispecific antibody significantly reduced mortality at a lethal doxorubicin dose and prevented body weight loss at a sublethal dose.
Bispecific antihapten anticancer ADCx^39^	Figure 1B	Tetravalent bispecific IgG antibodies targeting tumor antigens linked to disulfide-stabilized antidigoxigenin scFvs that binds digoxigenin.	*K*_D_ (for digoxigenin): 15.8 nM DAR: 2	In vitro: Target cell–specific delivery and internalization of digoxigeninylated payload In vivo biodistribution/PK: Tumor-specific accumulation of digoxigeninylated payloads in HER2^+^ or IGF1R^+^ tumor xenografts in mice. No signal in antigen-negative tumors, or with uncomplexed digoxigeninylated payloads. In vivo toxicity: NA
Antibody complexed with protein A/G-drug conjugate^40^	Figure 1C	Cytotoxic payloads conjugated to PEGylated Fc-binding proteins (protein A/G), noncovalently complexed to IgGs.	*K*_D_ (for protein A/G-conjugate with IgG Fc): 16–21 nM DAR: 1–1.6	In vitro: IC_50_ in HER2^+^ cells was ∼16× lower than that in HER2^−^ cells for protein A/trastuzumab-based ADCx In vivo biodistribution/PK: NA In vivo toxicity: NA
MAGNET ADCs^41^	Figure 1D	Self-assembling ADCs using gemcitabine-conjugated MEP-based IgG affinity ligands.	*K*_D_ (for MAGNET-fluorescein with IgGs): 5–8 *μ*M DAR: 6	In vitro: IC_50_ in antigen^high^ cell lines was ∼4–16× lower than that in antigen^low^ cell lines. In vivo biodistribution/PK: Gemcitabine concentration in mice xenograft tumors was not directly measured. Gemcitabine-cetuximab MAGNET ADC showed ∼2× tumor growth inhibition than gemcitabine + cetuximab combination. In vivo toxicity: No significant changes in the body weight, hematology, and liver/kidney functions were observed.
Aptamer-hapten ADCx (DOligobody)^42^, ^43^, ^44^	Figure 1E	Antihapten antibody complexed with a hapten-aptamer-payload conjugate. The aptamer targets specific cancer cells.	*K*_D_: not specified DAR: 2	In vitro: PAp7T8-DOligomer (aptamer-gemcitabine conjugate) significantly inhibited the viability of CFPAC-1 pancreatic cancer cells and PDOX-derived organoids in a dose-dependent manner. No in vitro studies were carried out using the full DOligobody. In vivo biodistribution/PK: DOligomer localized in tumor 15 minutes after administration. The DOligomer alone had a half-life of 0.02 h, while the DOligobody had a half-life of 29 h. In vivo toxicity: No significant changes in the body weight, hematology, and liver/kidney functions were observed.
Supramolecular ADCs^45^	Figure 1F	Anticancer antibody conjugated to a supramolecular “host” (calix[4]arene) encapsulating a cytotoxic “guest” molecule (doxorubicin). Drug release triggered by hypoxia.	*K*_D_ (for doxorubicin-calix[4]arene host-guest interaction): 256 nM DAR: 3.8	In vitro: Under normoxic conditions, the ADCx exhibited significantly reduced cytotoxicity compared with free doxorubicin. Under hypoxic conditions, its cytotoxicity increased and approached that of free doxorubicin. In vivo biodistribution/PK: Doxorubicin accumulation was ∼1.9–2.8× higher in the tumor in mice receiving the ADCx. In vivo toxicity: No significant changes in body weight, hematology, or serum enzyme tests. Histological studies showed no signs of organ damage or inflammation.
PROxAb shuttle^46^	Figure 1G	Anticancer antibodies linked to nanobodies targeting the E3-ligase ligands of PROTAC molecules.	*K*_D_ (for MIC7 nanobody-PROTAC binding): 419 pM to 8.3 nM DAR: 2	In vitro: IC_50_ in antigen-positive cell lines was ∼12–385× lower than in antigen-negative cell lines. Significant internalization of the ADCx was observed in antigen-positive cells within 1 h with negligible internalization in antigen-negative cells. In vivo biodistribution/PK: Half-life of GNE987 (PROTAC) increased from ∼2 (free) to ∼105 h (complexed), and the area under the curve increased by ∼236×. In vivo toxicity: No significant changes in body weight or adverse effects, even with repeat dosing.

## 2 ADCx formats

### 2.1 Bispecific antidrug anticancer ADCx

In 1994, Morelli et al^38^ used a hybrid hybridoma technique to produce DOXER2, a bispecific antibody capable of simultaneously binding doxorubicin and the epidermal growth factor receptor (EGFR) (Fig. 1A). In cellular cytotoxicity assays, DOXER2 decreased the IC_50_ of doxorubicin in EGFR-overexpressing A431 cells from 0.44 to 0.08 *μ*M and increased the IC_50_ of doxorubicin in EGFR-negative MeWo cells from 0.30 to 0.63–1.15 *μ*M. Administration of 20 *μ*g DOXER2 to A431-xenografted nude mice for 10 days, followed by the administration of a single dose of doxorubicin, significantly increased the accumulation of doxorubicin in tumors and reduced the concentration of doxorubicin in the intestine, heart, and kidneys. At lethal doses of doxorubicin (16 mg/kg), DOXER2 was able to significantly reduce mouse mortality. Finally, administration of 1 mg DOXER2, 24 hours before doxorubicin administration in A431-xenografted mice, produced tumor inhibition comparable to doxorubicin administered alone.

These studies demonstrated that DOXER2 did not impair the therapeutic efficacy of doxorubicin but reduced its toxicity. The results are consistent with DOXER2 reducing the cellular permeability of doxorubicin, enabling selective uptake in EGFR-expressing cells. Despite an increased tumor concentration of doxorubicin with DOXER2 co-administration, there was an equivalent antitumor response relative to the doxorubicin-only control group. There are several potential explanations for this surprising observation. First, doxorubicin release requires lysosomal catabolism of DOXER2 following EGFR-mediated endocytosis. Doxorubicin is a weakly basic drug, and as a result, can become positively charged and sequestered in the lysosome.^47^ Therefore, DOXER2 may have increased total doxorubicin delivery, but this benefit was partially negated by lysosomal sequestering. Additionally, the increased tumor concentration may represent doxorubicin bound to intact DOXER2 that had not undergone lysosomal catabolism. Unfortunately, only one concentration time point was examined, limiting a comprehensive understanding of the impact of DOXER2 on doxorubicin pharmacokinetics in both tumor and off-target tissues.

To our knowledge, DOXER2 was the first ADCx reported. Given the limited bispecific antibody engineering capability and a lack of potent payloads in the 1990s, this ADCx format may have been ahead of its time. The use of doxorubicin, which has relatively weak potency, limited the potential of the strategy. For example, several doxorubicin ADCs have been developed and evaluated in clinical trials; however, these ADCs were discontinued owing to limited clinical activity.^8^ Surprisingly, this format has not been evaluated with current ADC payloads and bispecific antibody production methods. Modern ADCs payloads (eg, monomethyl auristatin E [MMAE], DM4, pyrrolobenzodiazepine dimers, and PNU-159682) are ∼1000× more potent than doxorubicin.^48^ Revisiting this strategy with current-generation payloads and engineering platforms may result in a compelling extension from the original ADCx format.

### 2.2 Bispecific antihapten anticancer ADCx

**Fig. 2:**
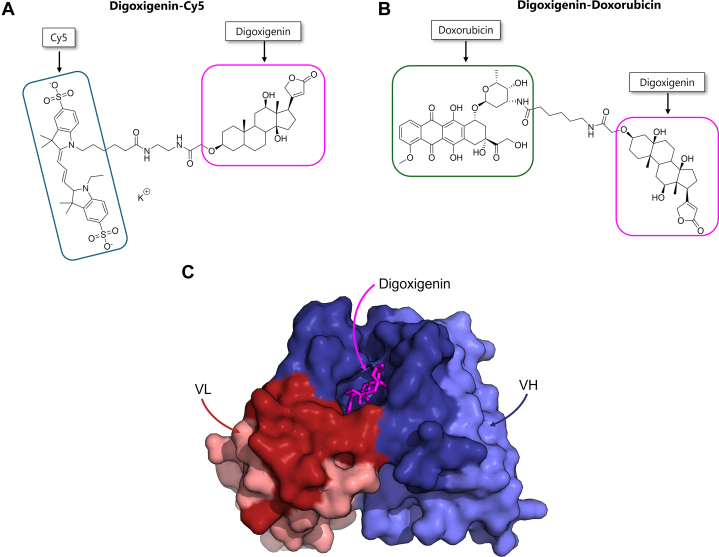
Structures of digoxigenin-conjugated payloads: (A) digoxigenin-Cy5 and (B) digoxigenin-doxorubicin (made using ChemBioDraw Ultra 14.0). (C) Structure of the antidigoxigenin antibody variable domains (VH and VL) complexed with digoxigenin (magenta). The complementarity determining regions of the heavy and light chains are shown in deep blue and red, respectively. The framework regions of the VH and VL are shown in light blue and pink, respectively. (From PDB 3RA7, made using PyMOL.) As seen in the figure, digoxigenin is buried deep in the antidigoxigenin scFv pocket. To extend the payloads above the scFv surface, flexible linkers of ∼15–20 Å having a chain length of 10–13 were used.

Metz et al from Roche^39^ reported a modular ADCx format using tetravalent bispecific antibodies. The bispecific antibodies were formatted as intact IgG fused with antidigoxigenin single-chain variable fragments (scFvs) on the C-termini of each IgG heavy chain through a flexible glycine-serine peptide linker (Fig. 1B). Digoxigenin was chosen as a hapten that could be conjugated to small payload molecules using commercially available digoxigenin conjugation kits. Digoxigenin conjugation enables the use of the same antidigoxigenin scFv for the delivery of multiple payloads. To demonstrate this, the authors conjugated doxorubicin and cyanine5 (Cy5) to digoxigenin (Fig. 2, A and B). The conjugation of a payload to digoxigenin did not alter the scFv binding affinity for digoxigenin. The authors also demonstrated that digoxigenin-conjugated payloads could be complexed with the bispecific antibodies by incubating for 10 minutes at room temperature.

For in vitro studies, the authors used the MCF-7 breast cancer cell line, which expresses intermediate levels of HER2 and insulin-like growth factor 1 receptor (IGF1R) and high levels of the carbohydrate antigen Lewis Y. Bispecific antibodies targeting Lewis Y, IGF1R, or HER2 were complexed with digoxigenin-Cy5 and incubated with MCF-7 cells. Flow cytometry revealed fluorescence only when digoxigenin-Cy5 was delivered via antigen-specific bispecific antibodies. In contrast, free digoxigenin-Cy5 or complexes using nontargeting antibodies yielded no significant signal, confirming antigen-dependent cellular targeting.

In vivo evaluation was performed using severe combined immunodeficient mice bearing xenografts of HER2^+^ (KPL-4 and Calu3) and IGF1R^+^ (H322M) tumor models. Mice were intravenously injected with bispecific antibodies complexed with digoxigenin-Cy5 or digoxigenin-doxorubicin, and payload distribution was monitored by near-infrared fluorescence imaging. In H322M tumors, IGF1R-targeting complexes showed rapid tumor-specific accumulation of digoxigenin-Cy5 within 30 minutes, with retention observed for up to 4 hours. Similarly, HER2-targeted digoxigenin-Cy5 complexes localized to KPL-4 tumors, with fluorescence detectable 24 hours postinjection. Control animals receiving free payloads exhibited no tumor-associated signal, confirming antibody-mediated targeting. The authors also evaluated a pretargeting approach, administering HER2-targeted bispecific antibodies 48 hours before digoxigenin-Cy5 injection. Imaging showed that digoxigenin-Cy5 selectively accumulated in HER2-positive tumors only in pretargeted mice, demonstrating in vivo assembly of ADCx.

This work provided a compelling proof of concept for an ADCx format using a hapten-payload strategy. Key strengths include the flexibility of using a single antihapten scFv to deliver various payloads, as well as the demonstration of both precomplexed and sequential administration formats. However, the approach has limitations. While digoxigenin conjugation enables modular payload design, it still requires chemical conjugation and linker optimization tailored to each payload to preserve bioactivity. Additionally, the authors used noncleavable linkers for conjugating doxorubicin and Cy5 to digoxigenin, which may limit intracellular drug release. The use of cleavable linkers would enable traceless payload release and mitigate the impact of digoxigenin on the pharmacology of the conjugated drug. Unfortunately, no in vivo efficacy studies were reported. Notably, the confocal microscopy images suggest that the payload becomes trapped in endolysosomal compartments following internalization, raising concerns about whether the relatively large digoxigenin conjugates can escape into the cytosol or nucleus to exert their intended biological effect. For payloads like doxorubicin that require nuclear access to intercalate DNA,^49^ lysosomal sequestration significantly reduces therapeutic efficacy.^47^

A significant limitation of the study is the lack of pharmacokinetic data regarding the biodistribution of the bispecific antibody, free payload, and payload-antibody complex. To minimize off-target payload release and maximize on-target drug delivery, high-affinity antihapten binding is required. The antidigoxigenin antibody used exhibits a moderate affinity (equilibrium dissociation constant [*K*_D_] = 15.8 nM), which likely results in a majority of the digoxigenin payload dissociating from the bispecific antibody in circulation. The dissociated hapten-payload conjugate may distribute into off-target tissues, rebind the bispecific antibody, or be cleared. The relative contribution of each pathway is dependent on the individual rates for each. For a slow-clearance drug with low permeability, moderate binding affinity may be sufficient to maintain ADCx concentrations. However, for compounds with fast plasma clearance and/or high tissue permeability, a high affinity (*K*_D_ < 100 pM) may be necessary to maintain ADCx concentrations over time.

### 2.3 Antibody complexed with protein A/G-drug conjugate

Protein A (SpA) and protein G (SpG) are proteins derived from *Staphylococcus aureus* and *Streptococcus* spp, respectively.^50^^,^^51^^,^^52^ They bind to the fragment crystallizable (Fc) regions of IgGs at the hinge region connecting the CH_2_ and CH_3_ domains, with a *K*_D_ of ∼10^−7^–10^−8^ M.^52^, ^53^, ^54^ Maso et al^40^ used the binding interactions between SpA or SpG and IgG Fc to develop noncovalent ADCx (Fig. 1C). Both SpA and SpG were conjugated to Cy5 or tubulysin A via 5- and 20-kDa polyethylene glycol (PEG) linkers. A PEG linker was used, instead of direct conjugation, to improve the hydrophilicity of the conjugates,^55^ decreasing the formation of precipitates on binding with the IgG Fc. PEGylated SpA and SpG showed a molar complexation ratio of 1.6 and 1 with rituximab and trastuzumab, respectively. While PEGylation reduced the molar binding ratio, possibly due to steric hindrance, it did not significantly affect the binding affinities.

To evaluate the binding specificity of the ADCx systems to target antigens, Cy5-PEG5kDa-SpA/rituximab for CD20-expressing cell lines and Cy5-PEG20kDa-SpG/trastuzumab for HER2-expressing cell lines were studied. The Cy5-PEG5kDa-SpA/rituximab ADCx bound to CD20-positive BL-41, Raji, and lymphoblastoid cell lines, with fluorescence intensity comparable with that of free rituximab. Similarly, Cy5-PEG20kDa-SpG/trastuzumab exhibited strong binding to HER2/neu-positive SKOV3 and SKBR3 cells, matching the binding profile of free trastuzumab. Both ADCx showed no interactions with antigen-negative cells. The addition of nonspecific antibodies did not impact the cell bound fluorescent signal, indicating there was minimal transfer of protein A/G during a 30-minute incubation. The therapeutic efficacy of tubulysin A-PEG20kDa-SpG/trastuzumab was investigated by evaluating their in vitro cytotoxicity against HER2/neu-positive and HER2/neu-negative cancer cell lines. In HER2/neu-positive SKBR3 cells, the complexes exhibited an IC_50_ of 0.006 *μ*g/mL. In contrast, the cytotoxicity was significantly reduced in HER2/neu-negative MDA-MB-231 cells, yielding an IC_50_ of 0.101 *μ*g/mL. Free tubulysin A exhibited comparable cytotoxic effects in both cell lines (SKBR3, IC_50_ = 0.045 *μ*g/mL; MDA-MB-231, IC_50_ = 0.085 *μ*g/mL), highlighting the selective efficacy of the ADCx in targeting HER2/neu-positive cells.

An advantage of this approach is that the same payload-SpA or payload-SpG conjugate can be complexed with different cancer-targeting antibodies, enabling high throughput screening of targeting antibodies. However, some limitations must be noted. This system still relies on 2 covalent conjugations: first to link PEG to SpA/SpG and then to attach the drug to the PEG moiety. These chemical reactions must be optimized separately for each payload, which may hinder scalability and increase development complexity. In addition, while PEGylation is commonly used to shield immunogenic epitopes,^55^ the use of bacterial proteins such as SpA and SpG still carries the risk of immunogenicity.^56^ The study does not conduct any immunogenicity studies to show a reduction in immunogenic responses to SpA/SpG after PEGylation. The authors also did not provide any data on the pharmacokinetics or in vivo stability of the antibody SpA/SpG-payload complexes. Finally, SpA/SpG bind to the same Fc region as the neonatal Fc receptor,^52^^,^^57^, ^58^, ^59^ potentially inhibiting Fc-recycling of the ADCx and, as a consequence, increasing the rate of nonspecific clearance.

### 2.4 Multivalent and affinity-guided antibody empowerment technology ADCs

4-mercaptoethylpyridine (MEP) is a small molecule that binds antibodies through hydrophobic interactions, van der Waals interactions, and hydrogen bonding.^60^ Gupta et al^41^ exploited these noncovalent interactions to develop ADCx by using MEP conjugates called multivalent and affinity-guided antibody empowerment technology (MAGNET) linkers. These MAGNET linkers were conjugated to gemcitabine and 5(6)-carboxyfluorescein, via cleavable amide bonds to generate MAGNET-gemcitabine and MAGNET-fluorescein, respectively (Fig. 1D). PEG was chosen as a linker between MEP and the payloads to increase hydrophilicity and prolong the circulation half-life of the MAGNET conjugates.^55^

The authors used molecular docking and molecular dynamics simulations to determine the binding sites of the MAGNET conjugates to IgG1 (cetuximab and trastuzumab). They identified 6 MAGNET-conjugate binding sites in each antibody analyzed. The complexation of MAGNET-gemcitabine and MAGNET-fluorescein with the antibodies was achieved by incubation of the 2 components at a pH of 8.5 at 50 °C for 8 minutes. Isothermal titration calorimetry was used to confirm the 1:6 antibody to MAGNET-conjugate stoichiometry. The 1:6 ratio was constant for both antibodies and both conjugates (MAGNET-gemcitabine and MAGNET-fluorescein).

In vitro studies were conducted using fluorescence microscopy to visualize the internalization of trastuzumab complexed with MAGNET-fluorescein, in HER2-positive SKOV3 cells compared with that in HER2-negative T47D cells. The results showed internalized fluorescence in SKOV3 cells, while T47D cells exhibited minimal uptake. Evaluation of the cytotoxic potential of MAGNET ADCs was performed using cetuximab-MAGNET-gemcitabine and trastuzumab-MAGNET-gemcitabine, which target EGFR and HER2, respectively. Both MAGNET ADCs were analyzed in cell lines expressing high and low levels of their corresponding target antigens. The IC_50_ for cetuximab-MAGNET-gemcitabine was 16.8-fold lower in HCT 116 cells and 4.2-fold lower in A549 cells than that in the EGFR-low SW620 cell line. Similarly, trastuzumab-MAGNET-gemcitabine exhibited a 13.3-fold lower IC_50_ in HER2-high SKOV3 cells than that in HER2-low T47D cells.

The efficacy of MAGNET ADCs was further investigated using a xenograft mouse model of human lung adenocarcinoma. Mice were treated with either cetuximab-MAGNET-gemcitabine or cetuximab alone at a dose of 20 mg/kg, administered intravenously every 4 days for 10 doses. Tumor growth was monitored over 34 days. A tumor growth inhibition of 63.1% was observed for cetuximab-MAGNET-gemcitabine compared with that of 32.5% for cetuximab alone. Toxicology studies in mice indicated that there was no significant change in toxicity biomarkers with cetuximab-MAGNET-gemcitabine administration up to 50 mg/kg. Finally, the plasma concentrations of cetuximab-MAGNET-fluorescein were similar to total cetuximab concentrations over a 20-day period.

The MAGNET ADC platform is a modular approach for generating ADCx using noncovalent affinity ligands. A strength of this platform is the generation of homogeneous complexes with DAR of 6. The “plug-and-play” nature of this system is particularly attractive as it enables rapid assembly of ADCx by simple incubation of off-the-shelf antibodies with presynthesized MAGNET-payload conjugates, thereby allowing the same linker-payload construct to be complexed with antibodies targeting different antigens. Nonetheless, conjugating payloads to the MEP-based MAGNET linker requires chemical optimization and suitable reactive groups for each payload.

The pharmacokinetic study for MAGNET ADC used ELISA to determine total cetuximab concentrations using EGFR capture and MAGNET-fluorescein concentrations using an antifluorescein capture. One limitation of this analytical strategy is the inability of the ELISA to discern an antibody with a DAR of 1 from a DAR of 6. Therefore, it is difficult to determine the true loss of complexed payload over time. The authors reported that the equilibrium dissociation constant for MAGNET-fluorescein to cetuximab was ∼18 *μ*M. This binding affinity is consistent with rapid dissociation (binding half-life of minutes), which raises concerns about off-target payload release, rapid payload clearance, or possible transfer of the MAGNET conjugate to endogenous antibodies. Additionally, the authors observed a different binding affinity for MAGNET conjugates to antigen-binding fragments vs intact antibodies, indicating there may be heterogeneity in the release rate of MAGNET conjugates from the individual binding sites.

### 2.5 Aptamer-hapten ADCx

Aptamers are short, single-stranded oligonucleotides that fold into defined 3-dimensional structures capable of binding to cellular targets with high specificity and affinity.^61^ Their small size, low immunogenicity, and ease of synthesis and modification make them attractive alternatives to antibodies in targeted therapy.^62^ However, owing to their small size and hydrophilicity, aptamers are rapidly cleared by renal filtration.^62^^,^^63^ To overcome these limitations, a novel platform termed oligobody was developed, in which a hapten-conjugated aptamer is noncovalently complexed with an antihapten antibody (Fig. 1E), thereby combining the targeting capability of aptamers with the favorable pharmacokinetics of antibodies.^42^, ^43^, ^44^

The first demonstration of this strategy was provided by Heo et al,^42^ who conjugated the antivascular endothelial growth factor aptamer t44-OMe to the hapten cotinine and complexed it with a chimeric anticotinine antibody. The complex exhibited significantly prolonged serum half-life compared with free aptamer and showed enhanced tumor penetration in a xenograft mouse model of lung cancer. Building upon this concept, the same group developed a therapeutic version of the oligobody termed the DOligobody, which incorporates a cytotoxic payload.^43^ An anti-HER2 aptamer was conjugated with cotinine and the microtubule inhibitor MMAE. This cotinine-HER2apt-MMAE construct, when complexed with the anticotinine antibody, selectively bound HER2^+^ NCI-N87 gastric cancer cells and effectively induced cytotoxicity. In vivo, systemic administration of the HER2-DOligobody significantly suppressed tumor growth in a HER2^+^ NCI-N87 xenograft model. Compared with PBS or control DOligobody groups, both HER2apt14-DOligobody and HER2apt28-DOligobody treatment groups showed a statistically significant reduction in tumor volume over the 34-day monitoring period. Tumor growth inhibition exceeded 60% by day 34 relative to the control group. Importantly, the treatment did not result in significant changes in body weight or serum biomarkers, suggesting a favorable toxicity profile.

Choi et al^44^ extended the DOligobody platform to pancreatic ductal adenocarcinoma (PDAC). Using a cell-SELEX (systematic evolution of ligands by exponential enrichment) approach, they identified a PDAC-specific aptamer (PAp7), which was subsequently optimized to PAp7T8 through truncation and chemical modification to enhance stability and cellular uptake. The final PAp7T8-DOligobody incorporated digoxigenin as the hapten and MMAE as the cytotoxic payload, forming a complex with a humanized antidigoxigenin antibody. In vitro, the complex demonstrated specific binding and internalization in PDAC cell lines and patient-derived organoids. In vivo, the DOligobody showed enhanced tumor accumulation, extended circulation time, and significant antitumor activity in orthotopic and patient-derived orthotopic xenograft models without causing systemic toxicity. This study did not report the specific target molecule for the aptamer, which impedes the interpretation of the data and the potential for translational relevance.

These results highlight the versatility of the DOligobody system in adapting to different cancer types and payloads by simply swapping the aptamer module. An advantage of this approach is that it strictly controls the DAR to 2. These works provide detailed pharmacokinetic and efficacy studies that demonstrate the advantages of using this approach compared with those of aptamer-drug conjugates alone. Despite its promising efficacy and modular design, several limitations of the oligobody/DOligobody strategy must be noted. Although aptamers can be rapidly synthesized and selected in vitro, the process of SELEX to generate highly specific aptamers remains labor intensive and must be repeated for each new target.^64^^,^^65^ Second, this approach still requires optimization of the covalent conjugation chemistry between the hapten and aptamer, and the payload and aptamer, such that their binding affinities and activity are not significantly impacted. Additionally, the aptamers used in the DOligobody studies have affinities (*K*_D_) between 15 and 20 nM, which are weaker than what can be achieved by using antibodies directly for targeting.^2^^,^^27^ Finally, it remains unclear what unique advantage an aptamer offers over an antibody for targeting cancer antigens. In the context of targeted drug delivery, particularly when an antibody is still required as part of the delivery scaffold (as in the DOligobody system), the justification for incorporating an aptamer rather than a traditional ADC is not clearly demonstrated.

### 2.6 Supramolecular ADCs

Supramolecular ADCs are composed of a targeting antibody conjugated to “host” molecules. These host molecules are macrocyclic compounds, such as pillar[n]arenes,^66^ calix[n]arenes,^67^ cucurbit[n]urils,^68^ and cyclodextrins.^69^ The host molecules, by their ring structure, can capture and noncovalently bind to a wide array of “guest” molecules.^70^ In case of supramolecular ADCs, these guest molecules can be either cytotoxic drugs or imaging agents that can behave as guests to the host molecules that are conjugated to the antibody (Fig. 1F). The advantage of this approach is that a single antibody-host conjugate can be used to noncovalently complex different drugs/imaging agents.

Wu et al^45^ conjugated maleimide-functionalized sulfonate azocalix[4]arenes (Mal-SAC4A host) to trastuzumab. The maleimide moiety allows the host to be conjugated to the antibody through reduced cysteines via click chemistry. The Mal-SAC4A bound 7 of the 10 tested guest molecules, including anticancer agents—doxorubicin, paclitaxel, NLG919, camptothecin, and methotrexate, and imaging agents/fluorophores—rhodamine B and cyanine 5-dimethyl, with a *K*_D_ of less than 10 *μ*M. These guest molecules, which showed affinity for Mal-SAC4A, included molecules with a wide range of hydrophobicity and hydrophilicity, demonstrating the versatility of the platform.

Cell-based studies using HER2-overexpressing SKBR3 breast cancer cells demonstrated specific binding of trastuzumab-Mal-SAC4A/Dox complex to tumor cells, as confirmed by confocal laser scanning microscopy. The azo moiety of Mal-SAC4A is reduced in hypoxic environments, such as the tumor microenvironment, due to the upregulation of bioreductases.^71^^,^^72^ Cytotoxicity assays demonstrated that the complex remained relatively nontoxic under normoxia with efficient payload release in hypoxic conditions, leading to enhanced cytotoxic effects against tumor cells.

Biodistribution studies in SKBR3 tumor-bearing BALB/c nude mice demonstrated preferential accumulation of trastuzumab-Mal-SAC4A/Dox in tumor tissues rather than major organs, confirming their tumor-targeting potential. The therapeutic efficacy of trastuzumab-Mal-SAC4A/Dox was assessed by administering the conjugates intravenously into SK-BR-3 tumor-bearing mice, with free doxorubicin, trastuzumab, and covalently conjugated trastuzumab-doxorubicin as controls. Tumor volume measurements over 21 days revealed significantly greater tumor suppression in mice treated with trastuzumab-Mal-SAC4A/Dox compared with those in other groups. Additionally, no significant weight loss or systemic toxicity was observed in treated mice, as confirmed by histopathological analysis and blood chemistry assays.

While the authors tested the stability of these complexes in the presence of blood components, it is possible that the payload guest molecule may be competitively displaced by endogenous compounds in the body or by coadministered drugs having higher affinity for the calix[4]arene, leading to inconsistent in vivo drug release profiles. The release mechanism relies on a reduction-triggered process under hypoxic conditions, but the variability of hypoxia in different tumor regions^73^^,^^74^ could result in uneven drug release or release in off-target tissues such as the bone marrow, which is physiologically hypoxic under normal conditions.^75^ The pharmacokinetic profiling in tumor-free mice showed that the circulation half-life of the trastuzumab-Mal-SAC4A/Dox was comparable with that of trastuzumab. However, the method used to assess circulation time (quantifying Cy5 fluorescence conjugated to the antibody) does not distinguish between intact trastuzumab-Mal-SAC4A/Dox and the antibody alone following dissociation of the payload. As Cy5 was conjugated to the antibody and not to the payload, any premature release or systemic clearance of doxorubicin would not be detected by this approach. Thus, the measured fluorescence signal only reflects the persistence of the antibody or antibody-calixarene construct and not necessarily the intact ADCx. This limits the conclusiveness of the claim regarding in vivo stability of the complete ADCx system, particularly with respect to payload retention. The long-term safety, immunogenicity, and off-target effects, particularly those due to the calix[4]arene moiety, also need to be thoroughly investigated. The calix[4]arene rings also significantly increase the hydrophobicity of the antibody, which can cause problems such as instability, aggregation, and off-target cytotoxicity.^28^^,^^29^

### 2.7 PROxAb shuttles

Proteolysis targeting chimeras (PROTACs) are a rapidly growing class of heterobifunctional small molecules.^76^ They are composed of 2 parts: a ligand that engages with a protein of interest and a ligand that binds to an E3 ligase, connected through a chemical linker. Once inside the cell, PROTACs bridge a protein of interest with an E3 ligase, prompting the E3 ligase to polyubiquitinate the protein. Polyubiquitination serves as a degradation signal for the cellular proteasome, which subsequently degrades the target protein.^77^ Two main E3 ligases are targeted: von Hippel-Lindau and cereblon. VH032 and its close derivatives are the most used von Hippel-Lindau ligands, while thalidomide and its close derivatives (including pomalidomide and lenalidomide) are the most used cereblon ligands.^78^ Therefore, a small number of E3 ligase ligands have been used in the hundreds of PROTAC molecules reported in current literature.^78^ This similarity in structures of the ligands, and their widespread use provides a unique opportunity to develop ADCx. A high-affinity binding partner, such as an antibody, scFv or nanobody that binds to a single E3 ligase ligand would enable a versatile delivery platform for all PROTACs derived from that E3 ligase ligand. To this effect, Schneider et al^46^ developed PROxAb shuttles. PROxAb shuttles are bispecific antibodies composed of targeting IgG antibodies, with their VH C-termini conjugated to an anti-VH032 single domain antibody (also known as VHHs or nanobodies) through a glycine-serine linker (Fig. 1G). The designed VH032-binding VHH named MIC7 was observed to have high binding affinity (*K*_D_ ∼1 nM) for most VH032 derivatives. The authors then complexed a VH032 containing BRD4-degrading PROTAC (termed GNE987) and a VH032-pHAb dye to the bispecific antibody. This was achieved by incubating both components (PROTAC or VH032-pHAb conjugate and the bispecific antibody) in a 2:1 ratio at room temperature.

Cell-binding studies with CD33^+^ MV4-11 cells demonstrated that the binding of the gemtuzumab-based PROxAb shuttles, complexed or noncomplexed with a PROTAC, was comparable with the parental unmodified antibody. The fusion of the MIC7 VHH domain did not impair the binding to the target cell surface receptors such as CD33. Flow cytometric analysis confirmed the internalization of the PROxAb shuttle into CD33^+^ cells. Complete BRD4 degradation was observed at PROxAb concentrations as low as 1 nM. Additionally, dose-response experiments showed that the PROxAb shuttles, complexed with PROTACs such as GNE987, exhibited nanomolar potencies in target-positive cells with minimal activity observed for nontargeting PROxAb shuttles or antigen-negative cells.

In vivo pharmacokinetic studies in mice revealed that the PROxAb shuttles significantly prolonged the half-life of complexed PROTACs. For example, GNE987’s half-life increased from 2 hours to over 4 days when complexed with the anti-CD33xMIC7 PROxAb shuttle. Additionally, the therapeutic potential of PROxAb shuttles was evaluated in MV4-11 xenograft models, where the complexed anti-CD33xMIC7 PROxAb shuttle exhibited superior antitumor efficacy than free PROTAC alone. The bispecific antibody remains in circulation following PROTAC dissociation and elimination. Therefore, the authors evaluated the administration of a chaser dose of free PROTAC. Re-dosing significantly prolonged the antitumor effect, demonstrating the potential for sequential dosing strategies to enhance the therapeutic outcome. This approach may be particularly beneficial for PROTACs that have no off-target toxicities but are limited by rapid clearance and poor oral bioavailability. For example, ASP3082, a selective KRAS G12D degrader currently being evaluated in clinical trials, is delivered as an infusion owing to poor oral bioavailability.^79^

To our knowledge, the aforementioned was the first study to describe the use of noncovalent antibody complexes for delivering PROTACs. This approach is particularly exciting because it does not require drug conjugation. A single VHH against an E3-ligase ligand can be used for multiple PROTACs using the same or similar E3-ligase ligands, eliminating the need for designing new binders for each drug. However, a drawback of this approach is that for every cancer antigen to be targeted, new vectors consisting of the sequences of new heavy chains linked to the VHH must be designed, transfected, and expressed. To overcome this limitation, in a follow-up study,^80^ the authors developed a chemoenzymatic approach that allowed for direct “welding” of the anti-PROTAC VHHs onto any antibody without the need for designing a new expression system. The authors used microbial transglutaminase (MTG), an enzyme that catalyzes the formation of isopeptide bonds between glutamine residues in proteins and acyl-acceptor substrates containing primary amines. The MTG targets the accessible glutamine 295 in the heavy chain of IgG1 antibodies. The VHHs are designed with an N-terminal triple glycine motif to act as an acyl acceptor. The reaction between the 2 moieties using MTG was conducted as a single step with incubation at 30 °C for 24 hours. Using the same PROTAC (GNE987), the authors showed that the welded PROxAb shuttles could be internalized in cells, and effectively degrade BRD4, preserving the characteristics of the original, recombinant PROxAb shuttles.

## 3 Perspectives and conclusions

The ADCx formats discussed in this review are under preclinical evaluation, and additional optimization and characterization are necessary. Because of the varied formats, broad conclusions regarding the potential of ADCx are difficult. Some formats still require covalent payload linkers and have exposed payload on the antibody surface. These formats are likely to share some limitations with traditional ADCs. However, a benefit is that antibody complex formation is simple, with some approaches (eg, MAGNET ADC) requiring only a 10-minute incubation with off-the-shelf antibodies. In contrast, traditional ADC synthesis involves multistep procedures (eg, reduction, conjugation, and purification), which can lead to increased antibody aggregation,^33^^,^^81^^,^^82^ heterogeneity,^26^^,^^32^^,^^83^ and production costs.^84^

The ADCx formats that directly bind to a drug (eg, bispecific antidrug ADCx and PROxAb shuttle) have unique attributes relative to ADCs. Direct drug binding obviates the need for linker-payload conjugation. This is particularly attractive for drugs without appropriate chemical handles for stable or specific linker conjugation. A secondary benefit of direct drug binding is the potential for mitigating drug exposure on the antibody surface. The binding mechanism of antidrug antibodies has been previously reviewed.^85^^,^^86^ Antidrug or antihapten antibodies wholly or partially conceal drugs in a binding pocket, as illustrated for digoxigenin in Fig. 2C. For hydrophobic drugs, decreased solvent exposure may minimize the increased hydrophobicity observed for traditional ADCs. Increased hydrophobicity has been reported to increase ADC clearance,^87^ payload release,^88^ ADC aggregation,^33^ and off-target binding.^89^ Furthermore, some drugs, such as tubulysin, have been reported to be metabolized when exposed on the ADC surface, leading to a rapid loss of activity in plasma.^90^ The limitation of antidrug binding is the need to identify an antidrug antibody for each payload. However, approved ADCs use a small number of payloads,^27^^,^^30^^,^^91^ therefore, a single antidrug antibody may be used to treat many cancers by modifying the specificity of the targeting arm. PROxAb shuttle avoids the requirement for many antidrug antibodies, as a single anti-E3 ligand antibody can be used to deliver hundreds of unique PROTACs.

A primary consideration in comparing ADCx with ADCs is the pharmacokinetics of the complex and free payload over time. For ADCs, owing to linker instability (eg, catabolism and maleimide exchange), the clearance rate of conjugated payload is several times faster than the antibody.^92^, ^93^, ^94^ Accelerated clearance increases plasma concentrations of free payload and decreases on-target drug delivery.^30^^,^^95^^,^^96^ It is counterintuitive that a noncovalent ADCx could decrease the amount of payload that is released off-target relative to a covalent ADC. For ADCx, the free concentration of payload is directly related to the antidrug antibodies’ binding affinity. Theoretically, with ultrahigh affinity, the half-life of drug dissociation can exceed the observed half-life of ADC linker cleavage. For example, Boder et al^97^ reported the development of an antifluorescein antibody with a dissociation half-life of 5.3 days, which is superior to the ∼4-day half-life observed in clinical trials for vcMMAE linker containing ADCs.^98^ In addition, antidrug antibody can rapidly rebind released payload,^46^ further minimizing off-target payload uptake, whereas ADC linker cleavage results in permanent payload release.

In conclusion, we believe ADCx is an underexplored drug delivery strategy. Although several exciting early-stage studies have been published, in-depth characterization and optimization efforts are necessary. Of particular interest would be a head-to-head comparison of a traditional ADC with an ADCx delivering the same payload. In some cases, ADCx may be ideally suited for the delivery of payloads that are unable to be used for ADCs due to a lack of functional groups for linker conjugation or poor in vivo stability when exposed on an antibody's surface. Taken together, ADCx represents a unique drug delivery strategy. With further research, ADCx has the potential to expand antibody-based drug delivery beyond traditional ADCs.

## Conflict of interest

The authors declare no conflicts of interest.

## References

[1] Slastnikova T.A., Ulasov A.V., Rosenkranz A.A., Sobolev A.S. (2018). Targeted intracellular delivery of antibodies: the state of the art. Front Pharmacol.

[2] Chau C.H., Steeg P.S., Figg W.D. (2019). Antibody–drug conjugates for cancer. Lancet.

[3] Parakh S., Lee S.T., Gan H.K., Scott A.M. (2022). Radiolabeled antibodies for cancer imaging and therapy. Cancers (Basel).

[4] Rybchenko V.S., Aliev T.K., Panina A.A., Kirpichnikov M.P., Dolgikh D.A. (2023). Targeted cytokine delivery for cancer treatment: engineering and biological effects. Pharmaceutics.

[5] Dugal-Tessier J., Thirumalairajan S., Jain N. (2021). Antibody-oligonucleotide conjugates: a twist to antibody-drug conjugates. J Clin Med.

[6] Marsh J.W., Srinivasachar K., Neville D.M. (1988). Antibody-toxin conjugation. Cancer Treat Res.

[7] Xenaki K.T., Oliveira S., van Bergen en Henegouwen P.M.P. (2017). Antibody or antibody fragments: implications for molecular imaging and targeted therapy of solid tumors. Front Immunol.

[8] Maecker H., Jonnalagadda V., Bhakta S., Jammalamadaka V., Junutula J.R. (2023). Exploration of the antibody-drug conjugate clinical landscape. MAbs.

[9] Wang R., Hu B., Pan Z. (2025). Antibody–drug conjugates (ADCs): current and future biopharmaceuticals. J Hematol Oncol.

[10] Udofa E., Sankholkar D., Mitragotri S., Zhao Z. (2024). Antibody drug conjugates in the clinic. Bioeng Transl Med.

[11] Liang Y., Zhang P., Li F., Lai H., Qi T., Wang Y. (2023). Advances in the study of marketed antibody-drug conjugates (ADCs) for the treatment of breast cancer. Front Pharmacol.

[12] Najminejad Z., Dehghani F., Mirzaei Y. (2023). Clinical perspective: antibody-drug conjugates for the treatment of HER2-positive breast cancer. Mol Ther.

[13] Riccardi F., Dal Bo M., Macor P., Toffoli G. (2023). A comprehensive overview on antibody-drug conjugates: from the conceptualization to cancer therapy. Front Pharmacol.

[14] Keam S.J. (2020). Trastuzumab deruxtecan: first approval. Drugs.

[15] Goto K., Goto Y., Kubo T. (2023). Trastuzumab deruxtecan in patients with *HER2*-mutant metastatic non–small-cell lung cancer: primary results from the randomized, phase II DESTINY-Lung02 trial. J Clin Oncol.

[16] Smit E.F., Felip E., Uprety D. (2024). Trastuzumab deruxtecan in patients with metastatic non-small-cell lung cancer (DESTINY-Lung01): primary results of the HER2-overexpressing cohorts from a single-arm, phase 2 trial. Lancet Oncol.

[17] Siena S., Di Bartolomeo M., Raghav K. (2021). Trastuzumab deruxtecan (DS-8201) in patients with HER2-expressing metastatic colorectal cancer (DESTINY-CRC01): a multicentre, open-label, phase 2 trial. Lancet Oncol.

[18] Raghav K., Siena S., Takashima A. (2024). Trastuzumab deruxtecan in patients with HER2-positive advanced colorectal cancer (DESTINY-CRC02): primary results from a multicentre, randomised, phase 2 trial. Lancet Oncol.

[19] Meric-Bernstam F., Makker V., Oaknin A. (2024). Efficacy and safety of trastuzumab deruxtecan in patients with HER2-expressing solid tumors: primary results from the DESTINY-PanTumor02 phase II trial. J Clin Oncol.

[20] Li B.T., Meric-Bernstam F., Bardia A. (2024). Trastuzumab deruxtecan in patients with solid tumours harbouring specific activating HER2 mutations (DESTINY-PanTumor01): an international, phase 2 study. Lancet Oncol.

[21] Li B.T., Smit E.F., Goto Y. (2022). Trastuzumab deruxtecan in *HER2*-mutant non–small-cell lung cancer. N Engl J Med.

[22] Vaz Batista M., Pérez-García J.M., Garrigós L. (2025). The DEBBRAH trial: trastuzumab deruxtecan in HER2-positive and HER2-low breast cancer patients with leptomeningeal carcinomatosis. Med.

[23] McPherson M.J., Hobson A.D. (2020). Pushing the envelope: advancement of ADCs outside of oncology. Methods Mol Biol.

[24] Peck M., Rothenberg M.E., Deng R. (2019). A phase 1, randomized, single-ascending-dose study to investigate the safety, tolerability, and pharmacokinetics of DSTA4637S, an anti-*Staphylococcus aureus* thiomab antibody-antibiotic conjugate, in healthy volunteers. Antimicrob Agents Chemother.

[25] Casi G., Neri D. (2015). Antibody–drug conjugates and small molecule–drug conjugates: opportunities and challenges for the development of selective anticancer cytotoxic agents. J Med Chem.

[26] Beck A., Goetsch L., Dumontet C., Corvaïa N. (2017). Strategies and challenges for the next generation of antibody–drug conjugates. Nat Rev Drug Discov.

[27] Parit S., Manchare A., Gholap A.D. (2024). Antibody-drug conjugates: a promising breakthrough in cancer therapy. Int J Pharm.

[28] Aoyama M., Tada M., Yokoo H., Demizu Y., Ishii-Watabe A. (2022). Fcγ receptor-dependent internalization and off-target cytotoxicity of antibody-drug conjugate aggregates. Pharm Res.

[29] Grairi M., Le Borgne M. (2024). Antibody–drug conjugates: prospects for the next generation. Drug Discov Today.

[30] Nguyen T.D., Bordeau B.M., Balthasar J.P. (2023). Mechanisms of ADC toxicity and strategies to increase ADC tolerability. Cancers (Basel).

[31] Jain N., Smith S.W., Ghone S., Tomczuk B. (2015). Current ADC linker chemistry. Pharm Res.

[32] Bross P.F., Beitz J., Chen G. (2001). Approval summary: gemtuzumab ozogamicin in relapsed acute myeloid leukemia. Clin Cancer Res.

[33] Gandhi A.V., Randolph T.W., Carpenter J.F. (2019). Conjugation of emtansine onto trastuzumab promotes aggregation of the antibody–drug conjugate by reducing repulsive electrostatic interactions and increasing hydrophobic interactions. J Pharm Sci.

[34] Chan S.Y., Gordon A.N., Coleman R.E. (2003). A phase 2 study of the cytotoxic immunoconjugate CMB-401 (hCTM01-calicheamicin) in patients with platinum-sensitive recurrent epithelial ovarian carcinoma. Cancer Immunol Immunother.

[35] Ladror D., Gu C., Tong V. (2024). Preclinical characterization of catabolic pathways and metabolism of ABBV-011, a novel calicheamicin-based SEZ6-targeting antibody-drug conjugate. Drug Metab Dispos.

[36] Shadid M., Bowlin S., Bolleddula J. (2017). Catabolism of antibody drug conjugates and characterization methods. Bioorg Med Chem.

[37] Dere R., Yi J.H., Lei C. (2013). PK assays for antibody–drug conjugates: case study with ado-trastuzumab emtansine. Bioanalysis.

[38] Morelli D., Sardini A., Villa E. (1994). Modulation of drug-induced cytotoxicity by a bispecific monoclonal antibody that recognizes the epidermal growth factor receptor and doxorubicin. Cancer Immunol Immunother.

[39] Metz S., Haas A.K., Daub K. (2011). Bispecific digoxigenin-binding antibodies for targeted payload delivery. Proc Natl Acad Sci U S A.

[40] Maso K., Montagner I.M., Grigoletto A., Schiavon O., Rosato A., Pasut G. (2019). A non-covalent antibody complex for the delivery of anti-cancer drugs. Eur J Pharm Biopharm.

[41] Gupta N., Ansari A., Dhoke G.V. (2019). Computationally designed antibody–drug conjugates self-assembled via affinity ligands. Nat Biomed Eng.

[42] Heo K., Min S.W., Sung H.J. (2016). An aptamer-antibody complex (oligobody) as a novel delivery platform for targeted cancer therapies. J Control Release.

[43] Kim H.J., Sung H.J., Lee Y.M. (2020). Therapeutic application of drug-conjugated HER2 oligobody (HER2-DOligobody). Int J Mol Sci.

[44] Choi S.I., Lee Y.S., Lee Y.M. (2023). Complexation of drug and hapten-conjugated aptamer with universal hapten antibody for pancreatic cancer treatment. J Control Rel.

[45] Wu X., Yao S., Huang Q. (2024). Antibody–calixarene drug conjugate: a general drug delivery platform for tumor-targeted therapy. ACS Appl Mater Interfaces.

[46] Schneider H., Jäger S., Könning D. (Published online September 29, 2023). PROxAb shuttle: a non-covalent plug-and-play platform for the rapid generation of tumor-targeting antibody-PROTAC conjugates. Preprint. bioRxiv.

[47] Kaufmann A.M., Krise J.P. (2007). Lysosomal sequestration of amine-containing drugs: analysis and therapeutic implications. J Pharm Sci.

[48] Yaghoubi S., Karimi M.H., Lotfinia M. (2020). Potential drugs used in the antibody–drug conjugate (ADC) architecture for cancer therapy. J Cell Physiol.

[49] Laginha K.M., Verwoert S., Charrois G.J.R., Allen T.M. (2005). Determination of doxorubicin levels in whole tumor and tumor nuclei in murine breast cancer tumors. Clin Cancer Res.

[50] Derrick J.P., Wigley D.B. (1992). Crystal structure of a streptococcal protein G domain bound to an Fab fragment. Nature.

[51] Graille M., Stura E.A., Corper A.L. (2000). Crystal structure of a *Staphylococcus aureus* protein A domain complexed with the Fab fragment of a human IgM antibody: structural basis for recognition of B-cell receptors and superantigen activity. Proc Natl Acad Sci U S A.

[52] Sauer-Eriksson A.E., Kleywegt G.J., Uhlén M., Jones T.A. (1995). Crystal structure of the C2 fragment of streptococcal protein G in complex with the Fc domain of human IgG. Structure.

[53] Choe W., Durgannavar T.A., Chung S.J. (2016). Fc-binding ligands of immunoglobulin G: an overview of high affinity proteins and peptides. Materials (Basel).

[54] Deisenhofer J., Jones T.A., Huber R., Sjödahl J., Sjöquist J. (1978). Crystallization, crystal structure analysis and atomic model of the complex formed by a human Fc fragment and fragment B of protein A from *Staphylococcus aureus*. Hoppe Seylers Z Physiol Chem.

[55] Gao Y., Joshi M., Zhao Z., Mitragotri S. (2024). PEGylated therapeutics in the clinic. Bioeng Transl Med.

[56] Gefen T., Vaya J., Khatib S. (2013). The impact of PEGylation on protein immunogenicity. Int Immunopharmacol.

[57] Ollier R., Wassmann P., Monney T. (2019). Single-step protein A and protein G avidity purification methods to support bispecific antibody discovery and development. MAbs.

[58] Pyzik M., Kozicky L.K., Gandhi A.K., Blumberg R.S. (2023). The therapeutic age of the neonatal Fc receptor. Nat Rev Immunol.

[59] Deisenhofer J. (1981). Crystallographic refinement and atomic models of a human Fc fragment and its complex with fragment B of protein A from *Staphylococcus aureus* at 2.9- and 2.8-.ANG. resolution. Biochemistry.

[60] Cheng F., Li M.Y., Wang H.Q., Lin D.Q., Qu J.P. (2015). Antibody–ligand interactions for hydrophobic charge-induction chromatography: a surface plasmon resonance study. Langmuir.

[61] Zhu G., Chen X. (2018). Aptamer-based targeted therapy. Adv Drug Deliv Rev.

[62] Sanjanwala D., Patravale V. (2023). Aptamers and nanobodies as alternatives to antibodies for ligand-targeted drug delivery in cancer. Drug Discov Today.

[63] Adachi T., Nakamura Y. (2019). Aptamers: a review of their chemical properties and modifications for therapeutic application. Molecules.

[64] Sefah K., Shangguan D., Xiong X., O’Donoghue M.B., Tan W. (2010). Development of DNA aptamers using Cell-SELEX. Nat Protoc.

[65] Zhuo Z., Yu Y., Wang M. (2017). Recent advances in SELEX technology and aptamer applications in biomedicine. Int J Mol Sci.

[66] Song N., Lou X.Y., Ma L., Gao H., Yang Y.W. (2019). Supramolecular nanotheranostics based on pillarenes. Theranostics.

[67] Zhou Y., Li H., Yang Y.W. (2015). Controlled drug delivery systems based on calixarenes. Chinese Chem Lett.

[68] Barooah N., Mohanty J., Bhasikuttan A.C. (2022). Cucurbituril-based supramolecular assemblies: prospective on drug delivery, sensing, separation, and catalytic applications. Langmuir.

[69] Păduraru D.N., Niculescu A.G., Bolocan A., Andronic O., Grumezescu A.M., Bîrlă R. (2022). An updated overview of cyclodextrin-based drug delivery systems for cancer therapy. Pharmaceutics.

[70] Ma X., Zhao Y. (2015). Biomedical applications of supramolecular systems based on host–guest interactions. Chem Rev.

[71] Liao C., Liu X., Zhang C., Zhang Q. (2023). Tumor hypoxia: FROM basic knowledge to therapeutic implications. Semin Cancer Biol.

[72] George Joy J., Sharma G., Kim J.C. (2024). Tailoring polymeric nanocarriers for hypoxia-specific drug release: Insights into design and applications in clinics. Chem Eng J.

[73] Michiels C., Tellier C., Feron O. (2016). Cycling hypoxia: a key feature of the tumor microenvironment. Biochim Biophys Acta.

[74] Chen Z., Han F., Du Y., Shi H., Zhou W. (2023). Hypoxic microenvironment in cancer: molecular mechanisms and therapeutic interventions. Signal Transduct Target Ther.

[75] Mirchandani A.S., Sanchez-Garcia M.A., Walmsley S.R. (2025). How oxygenation shapes immune responses: emerging roles for physioxia and pathological hypoxia. Nat Rev Immunol.

[76] Neklesa T.K., Winkler J.D., Crews C.M. (2017). Targeted protein degradation by PROTACs. Pharmacol Ther.

[77] Li K., Crews C.M. (2022). PROTACs: past, present and future. Chem Soc Rev.

[78] Bricelj A., Steinebach C., Kuchta R., Gütschow M., Sosič I. (2021). E3 ligase ligands in successful PROTACs: an overview of syntheses and linker attachment points. Front Chem.

[79] Park W., Kasi A., Spira A.I. (2024). 608O Preliminary safety and clinical activity of ASP3082, a first-in-class, KRAS G12D selective protein degrader in adults with advanced pancreatic (PC), colorectal (CRC), and non-small cell lung cancer (NSCLC). Ann Oncol.

[80] Lehmann T., Schneider H., Tonillo J. (2024). Welding PROxAb shuttles: a modular approach for generating bispecific antibodies via site-specific protein–protein conjugation. Bioconjug Chem.

[81] Wagner-Rousset E., Janin-Bussat M.C. (2014). Antibody-drug conjugate model fast characterization by LC-MS following IdeS proteolytic digestion. MAbs.

[82] Guo J., Kumar S., Chipley M. (2016). Characterization and higher-order structure assessment of an interchain cysteine-based ADC: impact of drug loading and distribution on the mechanism of aggregation. Bioconjug Chem.

[83] Hingorani D.V. (2024). An overview of site-specific methods for achieving antibody drug conjugates with homogenous drug to antibody ratio. Expert Opin Biol Ther.

[84] Bhushan A., Misra P. (2024). Economics of antibody drug conjugates (ADCs): innovation, investment and market dynamics. Curr Oncol Rep.

[85] Al Qaraghuli M.M., Palliyil S., Broadbent G., Cullen D.C., Charlton K.A., Porter A.J. (2015). Defining the complementarities between antibodies and haptens to refine our understanding and aid the prediction of a successful binding interaction. BMC Biotechnol.

[86] Al Qaraghuli M.M., Kubiak-Ossowska K., Ferro V.A., Mulheran P.A. (2021). Structural analysis of anti-hapten antibodies to identify long-range structural movements induced by hapten binding. Front Mol Biosci.

[87] Meyer D.W., Bou L.B., Shum S. (2020). An in vitro assay using cultured Kupffer cells can predict the impact of drug conjugation on in vivo antibody pharmacokinetics. Mol Pharm.

[88] Tumey L.N., Li F., Rago B. (2017). Site selection: a case study in the identification of optimal cysteine engineered antibody drug conjugates. AAPS J.

[89] Endo Y., Takeda K., Mohan N. (2018). Payload of T-DM1 binds to cell surface cytoskeleton-associated protein 5 to mediate cytotoxicity of hepatocytes. Oncotarget.

[90] Su D., Kozak K.R., Sadowsky J. (2018). Modulating antibody-drug conjugate payload metabolism by conjugation site and linker modification. Bioconjug Chem.

[91] Wang Z., Li H., Gou L., Li W., Wang Y. (2023). Antibody–drug conjugates: recent advances in payloads. Acta Pharm Sin B.

[92] Gordon M.R., Canakci M., Li L., Zhuang J., Osborne B., Thayumanavan S. (2015). Field guide to challenges and opportunities in antibody–drug conjugates for chemists. Bioconjug Chem.

[93] Wei C., Zhang G., Clark T. (2016). Where did the linker-payload go? A quantitative investigation on the destination of the released linker-payload from an antibody-drug conjugate with a maleimide linker in plasma. Anal Chem.

[94] Chuprakov S., Ogunkoya A.O., Barfield R.M. (2021). Tandem-cleavage linkers improve the in vivo stability and tolerability of antibody–drug conjugates. Bioconjug Chem.

[95] Hengel S.M., Topletz-Erickson A.R., Kadry H., Alley S.C. (2024). A modelling approach to compare ADC deconjugation and systemic elimination rates of individual drug-load species using native ADC LC-MS data from human plasma. Xenobiotica.

[96] Shah D.K., King L.E., Han X. (2014). A priori prediction of tumor payload concentrations: preclinical case study with an auristatin-based anti-5T4 antibody-drug conjugate. AAPS J.

[97] Boder E.T., Midelfort K.S., Wittrup K.D. (2000). Directed evolution of antibody fragments with monovalent femtomolar antigen-binding affinity. Proc Natl Acad Sci U S A.

[98] Li C., Zhang C., Li Z. (2020). Clinical pharmacology of vc-MMAE antibody-drug conjugates in cancer patients: learning from eight first-in-human phase 1 studies. MAbs.

